# Expression and Membrane Topology of *Anopheles gambiae* Odorant Receptors in Lepidopteran Insect Cells

**DOI:** 10.1371/journal.pone.0015428

**Published:** 2010-11-03

**Authors:** Panagiota Tsitoura, Evi Andronopoulou, Daniela Tsikou, Adamantia Agalou, Maria P. Papakonstantinou, Georgia A. Kotzia, Vassiliki Labropoulou, Luc Swevers, Zafiroula Georgoussi, Kostas Iatrou

**Affiliations:** 1 Insect Molecular Genetics and Biotechnology Group, Institute of Biology, National Centre for Scientific Research “Demokritos”, Athens, Greece; 2 Laboratory of Cellular Signaling and Molecular Pharmacology, Institute of Biology, National Centre for Scientific Research “Demokritos”, Athens, Greece; University of California Davis, United States of America

## Abstract

A lepidopteran insect cell-based expression system has been employed to express three *Anopheles gambiae* odorant receptors (ORs), OR1 and OR2, which respond to components of human sweat, and OR7, the ortholog of *Drosophila*'s OR83b, the heteromerization partner of all functional ORs in that system. With the aid of epitope tagging and specific antibodies, efficient expression of all ORs was demonstrated and intrinsic properties of the proteins were revealed. Moreover, analysis of the orientation of OR1 and OR2 on the cellular plasma membrane through the use of a novel ‘topology screen’ assay and FACS analysis demonstrates that, as was recently reported for the ORs in *Drosophila melanogaster*, mosquito ORs also have a topology different than their mammalian counterparts with their N-terminal ends located in the cytoplasm and their C-terminal ends facing outside the cell. These results set the stage for the production of mosquito ORs in quantities that should permit their detailed biochemical and structural characterization and the exploration of their functional properties.

## Introduction

In mammals, the family of odorant receptors (ORs) belongs to the superfamily of G protein-coupled receptors (GPCRs), which are characterized by the presence of seven transmembrane (7TM) domains [1]. By contrast, insect ORs, which are also predicted to contain 7TM domains [2], are not related to any other proteins including GPCRs. In fact, computational analyses predict that the membrane topology of insect ORs, exemplified by those of *Drosophila melanogaster*, is reversed relative to canonical GPCRs, with the N-terminus being intracellular and the C-terminus being extracellular [3], [4]. Experimental evidence to that effect has been provided in *Drosophila* where the reverse topology of at least some of the ORs of this organism in the natural environment of the olfactory neuron was clearly demonstrated [5]. Further studies involving expression of a member of the *Drosophila* OR family in *Drosophila* S2 cells revealed that the inverse orientation of that receptor also occurred in the expression system [6]. Although the inverse orientation has been assumed to be typical of all insect ORs, this remains to be formally demonstrated.

Functional studies carried out to date on insect ORs including those of the mosquito *Anopheles gambiae* have been mainly performed using *in vitro* expression in *Xenopus* oocytes [7], [8] or the ‘empty neuron’ system of *Drosophila* [9], [10], [11], [12]. With these approaches substantial progress has been made in assessing receptor expression and determining ligand specificities, thus setting the stage for investigations on the mechanisms of OR signal transduction, which have yet to be resolved unequivocally [13], [14], [15]. These and previous genetic analyses in flies have also established that the functional insect OR consists of a heteromeric complex of unknown stoichiometry, with ORx/OR83b being the essential molecular unit of olfactory perception [5]. Despite this progress, however, little is still known about the structural details and structure-function relationships of the members of this novel family of transmembrane proteins. For mosquito ORs, in particular, despite the impressive progress that has been achieved recently on the front of receptor deorphanization [7], [9], their biochemical properties and architectural features including the details of their organization on the cell surface await elucidation. For the establishment of such properties, the employment of appropriate expression systems permitting the synthesis of larger quantities of the ORs is required.

Prominent amongst existing metazoan systems for efficient recombinant protein expression are those utilizing cell cultures derived from lepidopteran insect cells, either as hosts for baculovirus expression vectors [16] or as cell lines stably transformed with appropriate plasmid-based expression constructs [17]. The latter have the advantage of maintaining the integrity of the intracellular machinery for protein posttranslational modification and secretion and are considered superior to the baculovirus-based expression systems for production of secreted and plasma membrane-anchored proteins [17]. For efficient recombinant protein production in transfected and transformed lepidopteran cell lines, a highly efficient expression vector was developed [17], [18]. This was based on the activity of a strong cellular promoter of the domesticated silkmoth *B. mori*, which was further enhanced by two baculovirus genetic elements [19], [20], [21]. Using this system, high level expression was achieved for a number of secreted [22], [23], [24] and membrane-anchored proteins [25] including GPCRs [26], which were also shown to be fully functional.

In the current study we report on the use of lepidopteran insect cells for expressing three *A. gambiae* ORs, OR1, 2 and 7, as a prelude to the biochemical, structural and functional characterization of these and other mosquito ORs. OR1 and OR2 exhibit female-biased expression [27] and respond to components of human sweat, chemicals present in human emanations [7], [9], [28] and breeding sites, as does the *Culex* ortholog of OR2, CquiOR2, which was recently deorphanized and shown to be highly sensitive to indole, an oviposition attractant for *C. quinquefasciatus* [29]. OR7, on the other hand, is the ortholog of *Drosophila* OR83b sharing 78% amino acid identity with the latter [30] and considered to be essential for stabilization and trafficking of the other ORs in the olfactory neurons [31]. Using lepidopteran insect cells as an expression platform, efficient expression of mosquito ORs was achieved for the first time. In this system, OR2 appears to be forming homodimers, while both OR1 and OR2 form heterodimers with OR7. Finally, through the employment of a novel “topology assay” we demonstrate unequivocally that mosquito ORs are anchored on the plasma membranes of the expressing cells and have intracellular N-termini and extracellular C-termini.

## Materials and Methods

### Plasmid construction

**Figure 1:**
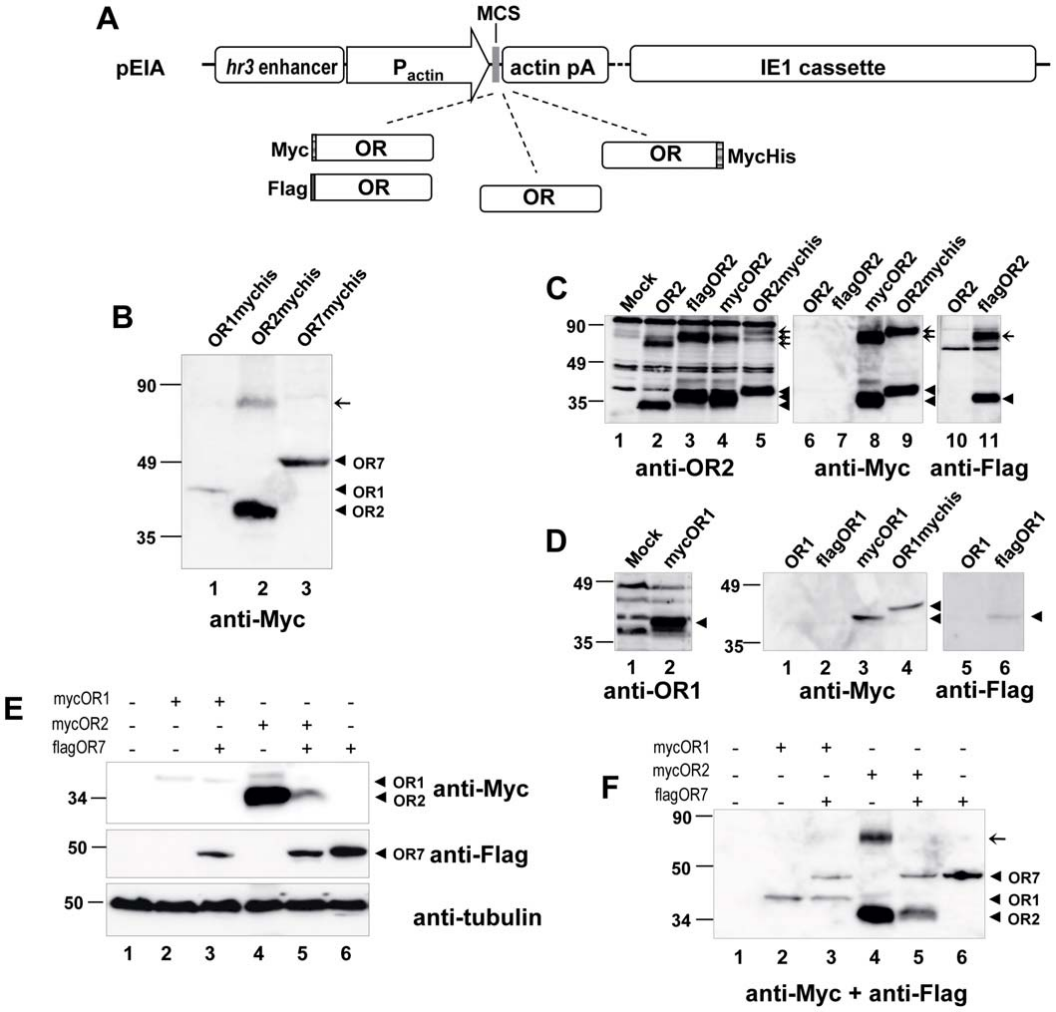
Expression of *A. gambiae* OR1, OR2 and OR7 in insect cells. (**A**) Schematic representation of the basic backbone vector (pEIA) used for the heterologous expression of various forms (tagged and untagged) of ORs in lepidopteran cells. *hr3* enhancer, baculoviral (BmNPV) homologous region 3 enhancer sequence; pActin, *Bombyx mori* A3 cytoplasmic actin promoter; MCS, multiple cloning site; actin pA, 3′untranslated region of *B. mori* actin gene containing polyadenylation signals; IE1 cassette, baculoviral (BmNPV) DNA fragment containing the *ie-1* transactivator gene under the control of its native viral promoter; OR; *A. gambiae* odorant receptor ORF; Myc, Flag and MycHis, epitope tags. (**B**) Detection of heterologous expression of C-terminally MycHis-tagged ORs in transfected Hi5 cells using Myc monoclonal antibody. (**C**) Detailed western blot analysis of OR2. Hi5 cells were transfected with plasmids expressing different versions of OR2, and lysates were analyzed using a specific polyclonal antibody against OR2 (left panel, lanes 1–5) or monoclonal antibodies against the Myc (middle panel, lanes 6–9) or the Flag epitope (right panel, lanes 10–11). Arrowheads and arrows indicate major bands corresponding to monomers and putative dimers, respectively. (**D**) Detailed western blot analysis of OR1. Hi5 cells were transfected with plasmids expressing different versions of OR1, and lysates were analyzed using monoclonal antibodies against the Myc (middle panel, lanes 1–4) or the Flag epitope (right panel, lanes 5–6). In the left panel immunoreactivity of the specific polyclonal antibody against OR1 is shown, with lysates from cells expressing mycOR1 after treatment with the proteasome inhibitor MG132. Molecular weight markers are shown on the left. (**E**) and (**F**) Effect of coexpression of OR7 on the expression levels of OR1 and OR2. Hi5 cells were transfected with constant amounts (45% of total DNA) of Myc-tagged OR1 or OR2, along with equal amounts of Flag-tagged OR7 or empty vector (pEIA), and pEIA-GFP (10% of total DNA, for evaluation of the efficiency of transfection). Whole cell lysates (**E**) and membrane fractions (**F**) were analysed by SDS-PAGE and western blot. Detection of OR1, OR2 and OR7 was done using the anti-Myc and anti-Flag antibodies either consecutively (in **E**) or simultaneously (in **F**). To control for loading, the whole lysate fractions were also probed with an anti-tubulin antibody.

Full-length coding sequences of *A. gambiae* odorant receptors (ORs) 1, 2 and 7 were amplified by PCR from an antennal cDNA library [32], using the oligonucleotide primer pairs OR1F/OR1R, OR2F/OR2R, and OR7F/OR7R, respectively (**Table 1**). For C-terminal epitope tagging of the receptors, the OR1SC, OR2SC and OR7SC oligonucleotides were instead used as reverse primers for PCR amplification. The OR coding sequences (417, 378 and 478 amino acids with predicted molecular masses of 48.5, 43.5 and 54 kDa, for OR1, OR2 and OR7, respectively; AnoBase and EnsemblMetazoa databases) were cloned into the expression vector pIE1/153A (henceforth pEIA, **Figure 1A**) [18], [20], [24] or in modified versions of the vector [17], which allow N-tagging with Flag (MDYKDDDDKD, molecular mass of 1.26 kDa) or Myc (MEQKLISEEDL, molecular mass of 1.33 kDa) epitopes, and C-terminal tagging with a *Xa*-Myc-6xHis (P*IEGR*SPVYSEQKLISEEDLPHHHHHH, molecular mass of 3.21 kDa) epitope (**Figure 1A**). The pEIA and pEA (which lacks the IE1 cassette) vectors were also used for the expression of fluorescent proteins when needed, as indicated. The convention employed for the identification of the location of the epitope tags on the fusion proteins was “tagORx” and “ORxtag”, for tags added at the NH2 and COOH termini, respectively, of the receptor proteins.

**Table 1:** List of oligonucleotides used in PCR. Restriction sites are underlined; initiation and termination codons are in bold and italics, respectively.

Primer	Sequence
OR1F	CCAGGATCCGAAAGTA**ATG**AAGCTGAAC
OR1R	CCAGGATCCA*TTA*CTCTGATTCCATGCT
OR1SC	CAAGGATCCCTCTGATTCCATGCTCTGAAG
OR2F	CAAAGATCTCACC**ATG**CTGATCGAAGAGTGTCC
OR2R	CCAAACAGATCTGT*TTA*GTTGTACACTCGGCG
OR2SC	CAACAGATCTGTTGTACACTCGGCGCAGC
OR7F	CCAAAGATCTCAGC**ATG**CAAGTCCAGCCGACCAAG
OR7R	CCACACAAGATCTGGCTGT*TTA*CTTCAGCTGCACC
OR7SC	CCACAAGATCTCTTCAGCTGCACCAGCACC
TevF	GGCGGATCCGGCCACCATGTCACTAGTGGAAAACCTGTATTTTCAGGGCCATCATCATCATC
TevR	CCTTCTAGACGGCCCTTCCATTGGCATATATTCCTCTTCATGATGATGATGATGATGGCCCTG
BamOR1F	GCCCGGGGGATCCGAAAGTA**ATG**ATGAAGC
SpeOR1R	CGGACTAGTGGTCCCTCTGATTCCATGCTCTGAAG
XbaOR1F	CATGTCTAGA **ATG**AAGCTGAACAAACTGAACCCA
NotOR1R	ACTGGCGGCCGC *TTA*CTCTGATTCCATGCTCTGAAG
BamOR2F	AGTCGGATCCCAAC**ATG**CTGATCGAAGAGTGTCCG
SpeOR2R	ACGTACTAGTGAGTTGTACACTCGGCGCAGCAG
XbaOR2F	AGTCTCTAGA **ATG**CTGATCGAAGAGTGTCCG
XbaOR2R	TTAATCTAGA *TTA*GTTGTACACTCGGCGCAGCAG
XbaHR3F	AGCTCTAGA **ATG**TTGAACATGTTTGATATGTGGAAC
NotHR3R	AATTGCGGCCGCCAA*TTA*TCCGTGCGTGTAATC
BamHR3F	AGCTGGATCCCAAC**ATG**TTGAACATGTTTGATATGTGG
SpeIHR3R	ACGTACTAGTCCGTGCGTGTAATCTAAAACAC
BamδORF	GGAGGGATCCG**ATG**GAACCGGCCCCCTCCGCC
SpeδORR	CGGAACTAGTGGTCCGGCGGCAGCGCCACCGCC
XbaδORF	GCCGTCTAGA **ATG**GAACCGGCCCCCTCCGCCGGC
XbaδORR	CCGGTCTAGATGGTCAGGCGGCAGCGCCACCGCC
TevProtFBamHI	GACTGGATCCCAAC**ATG**GGAGAAAGCTTGTTTAAGGGG
TevProtRBamHI	GACTGGATCCTTATTAGCGACGGCGAC

The constructs used for the “topology screening” assay expressing the ORs or the human δ-opioid receptor (δOR; [33], [34]) as translational fusion products with the HR3 transcription factor and the TEV cleavage site (OR1/OR2/δOR-THE-HR3 or HR3-THE-OR1/OR2/δOR) were made by ligation of PCR fragments (primers described in **Table 1**) corresponding to the above ORFs in-frame with a linker sequence consisting of the TEV cleavage site (ENLYFQG), a 6xHis site (HHHHHH) and an EE (Glu-Glu) epitope (EEEYMPME) (“THE” linker; LVENLYFQGHHHHHHEEEYMPMEGP, molecular mass of 3.1 kDa) using *Bam*HI-*Spe*I digests for the 5′-fusions and *Xba*I or *Xba*I-*Not*I digests for the 3′-fusions. The catalytic domain of TEV protease [35] was amplified from the pRK793 plasmid (Addgene) by PCR using the primers TevProtFBamHI and TevProtRBamHI (**Table 1**) and subsequently cloned into the *Bam*HI-site of the pEA vector. The GFP reporter construct (pBmbA/RORE.GFP), which consisted of the basal actin promoter of *B. mori* harboring four copies of the ROREdro element (*i.e.*, response element for retinoic acid receptor-related orphan receptor) in its upstream region, was based on the pBmbA/RORE.cat plasmid [36] after replacement of the *cat* gene with the GFP ORF. Finally, the pEIA-myc-µOR plasmid, used as positive control in FACS analysis, was obtained by subcloning a 1.2 kb *Eco*RV-*Xba*I fragment from cDNA3.1-myc-µOR (rat µ-opioid receptor, a gift of Dr. S. George, University of Toronto, Canada) into the *Sma*I-*Xba*I site of pEIA.

### Cell culture, transfection and transformation

*Bombyx mori* Bm5 [37] and *Trichoplusia ni* BTI-TN-5B1-4 HighFive™ cells [38] (thereafter indicated as Hi5) were grown in IPL-41 insect cell culture medium, supplemented with 10% fetal bovine serum (Life Technologies), were maintained at 28°C and subcultured weekly. Transfection was performed with Escort IV (Sigma) or Lipofectin (Invitrogen) reagents, according to standard protocols. For generation of stably transformed cell lines, expressing mycOR1, mycOR2 or OR2mychis, along with flagOR7, Bm5 cells were used because they originate from the same organism, the silkmoth *B. mori* (and its baculovirus, BmNPV), from which the control elements used for transgene expression were derived. These cells were cotransfected with the relevant expression plasmids (pEIA.OR) and pBmA.pac, a plasmid conferring resistance to puromycin, at molar ratios of 10∶1, using 5 µg of total plasmid DNA per 10^6^ cells. Stably transformed cell lines were maintained in IPL-41 supplemented with 10% FBS, 50 µg/ml gentamycin (Invitrogen) and 15 µg/ml puromycin. For transient expression, Hi5 cells were mainly used because of their better transfectability, thus higher levels of transient transgene expression, relative to Bm5 cells [39].

### Antibodies and Western blot analysis

Rabbit polyclonal antibodies against OR1 and OR2 were generated by IMGENEX (San Diego, USA) using the synthetic peptides NKLNPRWDAYDRRDS and DDIRPVLERYTRRGR encompassing residues 4–18 and 103–117 of OR1 and OR2, respectively. The OR-specific sera were used at a dilution of 1∶300–1∶500. For detection of the tagged forms, mouse anti-Myc (at 1∶1,000; Cell Signaling) and anti-Flag (at 1∶800; Sigma) antibodies were used. Anti-tubulin antibodies (at 1∶500) were from AbD Serotec. For transient expression experiments, 2.5×10^5^ Hi5 cells seeded in 24-well plates were transfected with plasmids containing different expression constructs of ORs. To assess transfection efficiencies, plasmids expressing fluorescent proteins were also included (10% of total DNA) in the transfection mixtures in some experiments. Cells were lysed with SDS-sample buffer (62 mM Tris-HCl pH 6.8, 2% SDS, 10% glycerol, 0.002% bromophenol blue) and sonicated. After addition of β-mercaptoethanol (5%), proteins were separated on 10 or 12% SDS-PAGE and electroblotted to Hybond-ECL nitrocellulose membrane (Amersham Pharmacia). Western blot analysis was performed either with the OR-specific antisera or the commercially available antibodies against the epitope tags, while secondary antibodies used were HRP-conjugated anti-rabbit IgG (Chemicon) and anti-mouse IgG (Santa Cruz), respectively (1∶1,000). For tubulin detection, the anti-rat secondary antibody (Chemicon, Millipore) was used (1∶1,000). Amersham ECL Western Blotting kit (GE Healthcare) or Pierce SuperSignal West Pico chemiluminescent substrate (ThermoScientific) were used for detection.

### Cell membrane preparations

Cells expressing the odorant receptors were harvested by centrifugation, resuspended in ice-cold buffer (10 mM Tris-HCl pH 7.5, 0.1 mM EDTA) and lysed by passing through a 1 ml syringe (27-gauge). Cell membranes were separated essentially as previously described [40]. Briefly, after low-speed centrifugation, the supernatant was collected and further centrifuged at 100,000×g for 30 min at 4°C and the resultant membrane fraction was resuspended in 10 mM Tris pH 7.5, 0.1 mM EDTA and stored at −70°C. For immunoblotting, 50–100 µg of proteins were analysed.

### Pull-down assays

Bm5 cells (7×10^6)^ transfected with plasmids expressing flagOR1, flagOR2, or OR7mychis, were solubilized in 2% dodecyl-β-d-maltoside, 50 mM Na-phosphate pH 8.0, 300 mM NaCl, 20 mM imidazole in the presence of a protease inhibitor cocktail (Sigma). Five hundred µg of cell lysates were mixed with 200 µl of 50% w/v Ni-NTA agarose (Qiagen) for 2 hours at 4^°^C. OR7mychis was initially bound on Ni-NTA agarose, followed by incubation with flagOR1 or flagOR2 to allow interaction with the immobilised protein. At the end of the incubation the mixture was washed extensively with 20 bed volumes of buffer containing 50 mM Na-phosphate pH 8.0, 300 mM NaCl, 20 mM imidazole. The Ni-NTA bound proteins were resolved by 10% SDS-PAGE and visualized by immunoblotting using the appropriate antibodies. Non-specific binding was assessed in parallel pull down assays employing mock-transfected Bm5 cell lysates.

### Immunofluorescence microscopy

Approximately 2×10^5^ of Bm5 or Hi5 cells expressing the tagged ORs, were seeded on poly-L-lysine coated microslides for 2 hours and subsequently fixed with 3.7% v/v formaldehyde and permeabilized, where necessary, with 0.1% v/v Triton X-100. After blocking for 1 h at room temperature with PBS containing 3% BSA the cells were incubated overnight with anti-Myc (1∶500) and/or anti- Flag (monoclonal or polyclonal, Sigma, at 1∶200) antibodies, using as secondary antibodies the anti-mouse FITC (Sigma) and anti-rabbit Alexa Fluor 568 (Molecular Probes). The cells were finally mounted with p-phenyl-diamine (optional staining with DAPI) and observed with a BioRad MRC-1024 laser scanning confocal microscope. To confirm the localization of recombinant ORs on the cellular plasma membrane, live Hi5 cells transfected with the relevant expression constructs were initially labeled with 5 µg/ml Texas Red-conjugated wheat germ agglutinin (WGA, Molecular Probes, Invitrogen) for 10 minutes in PBS at 28°C, followed by fixation and staining with anti-Myc primary (1∶700) and anti-mouse FITC secondary (1∶200) antibodies, in the presence or absence of 0.05% saponin (Fluka), and mounting with mowiol (Sigma). Confocal microscope data were processed using the GNU Image Manipulation Program.

### Topology screening assay

Hi5 cells were cotransfected with the GFP reporter construct (pBmbA/RORE.GFP) and the relevant fusion constructs (OR1/OR2/δOR-THE-HR3 or HR3-THE-OR1/OR2/δOR), together with the pEA.TEV plasmid or an empty expression vector. After two days, fluorescence was observed on a Zeiss (Jena, Germany) Axiovert 25 inverted microscope equipped with a HBO 50 illuminator for incident-light fluorescence excitation and a Zeiss filter set 09 (450–490 nm excitation filter, 510 nm barrier filter). For quantification, cells were harvested 48–72 h after transfection, washed once with PBS and lysed by three freeze/thaw cycles. After lysis, the fluorescence values were measured in the microplate reader Infinite M200 (Tecan Group Ltd).

### Fluorescent activated cell sorting (FACS) analysis

FACS analysis was performed essentially as described [41]. Briefly, Bm5 cells (10^6^ per sample) stably expressing N- or C-terminally tagged OR1 and OR2, in combination with flagOR7, were harvested and incubated overnight with the monoclonal anti-Myc antibody (Cell Signaling #9B11, 1∶750 dilution) at 4°C under rotation. Cells were subsequently washed with PBS containing 2% foetal bovine serum prior to 2 h incubation with an anti-mouse FITC conjugated secondary antibody (1∶100). Cells were extensively washed and fixed with 3% formaldehyde before analysis on a FACScan flow cytometer (Becton Dickinson Immunocytometry Systems, Inc). The mean fluorescence intensity of 6,000 cells was determined for each sample. As a positive control, Hi5 cells transfected with an analogous expression construct for a Myc-tagged GPCR, pEIA-myc-µOR, were similarly stained and subjected to FACS analysis.

## Results

### Expression of recombinant ORs in lepidopteran cells

The open reading frames (ORFs) of three *A. gambiae* odorant receptors, OR1, OR2 and OR7 (the *A. gambiae* homologue of *Drosophila* OR83b) were amplified from a female mosquito antenna cDNA library [32] and subcloned into different versions of the expression vector pEIA allowing expression of authentic, N-terminally or C-terminally tagged proteins (**Figure 1A**; [17]. This vector directs expression of the cloned ORFs at high levels due to double enhancement of the silkworm cytoplasmic actin promoter by the baculovirus (BmNPV) *hr3* enhancer and IE1 trans-activator [18], [20], [24].

The expression of all three ORs was initially examined in HighFive™ (Hi5) cells transfected with the C-terminally Myc-tagged constructs. As shown in **Figure 1B**, immunoblotting of whole cell extracts using a monoclonal antibody against the Myc epitope revealed the presence of three major immunoreactive species at approximately 43, 38.5 and 50 kDa, corresponding to the tagged versions of OR1, OR2 and OR7, respectively. From comparisons to the theoretical molecular masses of the three ORs (Materials & Methods), the expressed polypeptides migrated on SDS-PAGE somewhat faster than expected. Such a property has been reported previously for many other membrane proteins [42], [43] including some mammalian olfactory receptors [44].

The expression of the untagged and other tagged versions of the ORs in the Hi5 cells was also examined using OR-specific antibodies (for OR1 and OR2) and antibodies recognizing the epitope tags (for all expressed ORs). For OR2, the anti-Myc and anti-Flag antibodies specifically recognized the corresponding tagged forms (**Figure 1C**, middle and right panels), while the anti-OR2 antibody detected all four different forms of the receptor (in addition to a number of cross-reacting polypeptides that were also present in untransfected cells; **Figure 1C**, left panel). The relative migration of the detected polypeptides correlated well with the predicted sizes of the untagged and tagged versions of the protein [for size correlations see Materials & Methods].

Interestingly, for each form of recombinant OR2, another band of slower mobility whose size was increasing in proportion to the size of the tag could also be detected with all antibodies (**Figures 1B and 1C**). These forms may represent homodimers of OR2, as is the case with many other transmembrane proteins, which were reported to form dimeric complexes persisting in denaturing gel electrophoresis [44], [45], [46], [47].

An equivalent comparison was also performed for OR1. Again the anti-Myc and anti-Flag antibodies specifically recognized only the corresponding tagged forms (**Figure 1D**, middle and right panels, respectively), and in all cases the size of the detected polypeptides was in accordance to the sizes of the tags. Immunoreactivity with the specific antiserum generated against OR1 was also shown (**Figure 1D**, left panel), however the lower expression levels of OR1 and/or lower sensitivity of the polyclonal antibody did not allow a more thorough examination of this receptor with the specific antiserum, particularly with regard to the formation of putative homodimers.

Previous studies on the expression patterns of *D. melanogaster* have suggested that in the absence of OR83b, the constant heteromerization partner of a large number of *Drosophila* ORs, the ORs are highly unstable *in vivo* [5]. To determine whether co-expressing of OR7, the *A. gambiae* homologue of *Drosophila* OR83b, in this heterologous system affects the expression levels of OR1 and OR2, we transfected Hi5 cells with plasmids expressing N-terminally Myc-tagged OR1 or OR2, either alone or together with Flag-tagged OR7, and analyzed whole cell extracts and the membrane fractions of the expressing cells for the relative levels of expression of the recombinant proteins. As shown in **Figure 1E**, the western blots indicated that the expression levels of OR1 and OR2 in the whole cell extracts were not further enhanced upon expression of OR7, and thus did not appear to be dependent on the presence of this receptor. On the contrary, a decrease in the accumulation of all receptors could be observed, which might be attributed to competition due to endoplasmic reticulum overloading. Exactly the same situation was found to exist when the membrane fractions were analysed (**Figure 1F**).

### Subcellular localization of recombinant ORs and interaction with OR7

**Figure 2:**
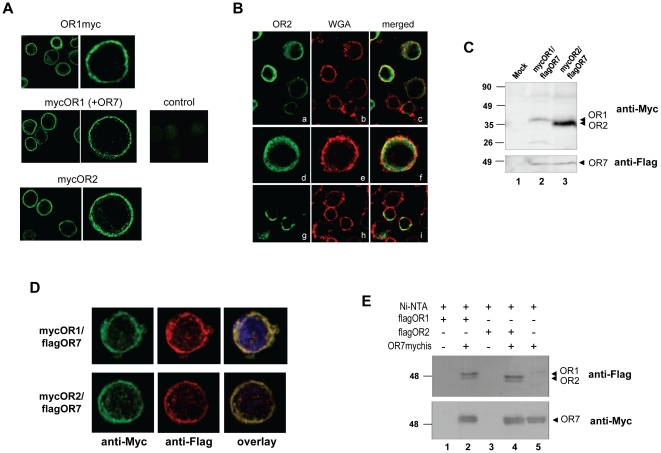
Co-localization of odorant receptors expressed in lepidopteran insect cells. (**A**) Expression constructs for N-terminally (mycOR1, mycOR2) or C-terminally tagged receptors (OR1myc) were transfected in Bm5 cells (in the absence/presence of OR7) and the localization of the expressed ORs was detected using anti-Myc antibody. Control indicates transfection with empty expression vector. (**B**) Co-localization of OR2 with the plasma membrane marker wheat germ agglutinin. Cells expressing OR2myc were double stained with WGA-Texas Red-X conjugate (b, e, f) and with anti-myc antibody (a, d, g) in the presence or absence of saponin (a-f and g-i, respectively). (**C**) Detection of ORs in the membrane fraction of stable cell lines coexpressing mycOR1 (lane 2) or mycOR2 (lane 3) along with flagOR7. Immunoblotting was performed with anti-Myc and anti-Flag antibodies (upper and lower panels, respectively). Membranes from Bm5 untransfected cells were used as a negative control (lane 1). (**D**) Co-localization of OR1 or OR2 with OR7. Bm5 cells were co-transfected with expression plasmids for N-terminally tagged mycOR1 or mycOR2 together with N-terminally tagged flagOR7 expression vector. Tagged ORs were detected with anti-Myc/anti-mouse fluorescein-labelled IgG and anti-Flag/anti-rabbit Alexa fluor-labelled IgG as indicated and counter-stained with DAPI. (**E**) Pull-down assays showing heteromerization between OR1 and OR7 or OR2 and OR7. Extracts containing C-terminally Myc-His-tagged OR7 were incubated with Ni^2+^-NTA beads and bound protein complexes were analyzed by Western blot by anti-Flag antibody (upper panel) for the presence of N-terminally Flag-tagged OR1 and OR2 or by anti-Myc antibody (lower panel) to detect OR7mychis.

To assess localization of the receptors, all available tagged constructs of ORs 1 and 2, alone or in combination with OR7, were transiently expressed in *B. mori* Bm5 cells. As illustrated by the representative images shown in **Figure 2A**, the immunofluorescence analyses revealed that the N- or C-terminally tagged receptors accumulate largely on the plasma membranes of the expressing cells and this localization is independent of the presence of OR7 or the position and type of the tag (N-terminal or C-terminal, Myc or Flag, respectively). Some intracellular fluorescence was also evident in the expressing cells. This was more prominent in the case of Hi5 cells, which are known to express recombinant proteins at higher levels relative to Bm5 cells [18], [39], upon transfection with the Myc-tagged OR2 expression construct (**Figure 2B**). Even in this case, however, counterstaining with wheat germ agglutinin demonstrated the anchoring of a major portion of the over-expressed receptor on the plasma membrane (**Figure 2B**). We hypothesize the presence of cytoplasmic ORs to be due to the inability of the endoplasmic reticulum/Golgi apparatus to process effectively all expressed receptors to the plasma membrane. Alternatively, the cytoplasmic ORs may simply reflect the presence of intermediates in receptor synthesis and membrane localization processing. Western blot analyses of cell membrane preparations from cell lines stably expressing mycOR1/flagOR7 or mycOR2/flagOR7 confirmed the presence of receptors 1, 2 and 7 in the membrane fraction (**Figure 2C** **;** also **Figure 1F** for transiently transfected cells**)**.

The colocalization of OR1 and OR2 with their presumed heteromerization partner, OR7, was confirmed by immunofluorescence assays employing secondary antibodies labelled with different fluorochromes. These experiments revealed overlapping fluorescent signals originating from the co-expressed receptor pairs, OR1/OR7 and OR2/OR7 (**Figure 2D**). To provide further evidence about the heteromerization of the OR1 and OR2 with OR7, pull-down assays were performed using whole lysates of cells transfected with expression constructs for the tagged versions of OR1 or OR2 and OR7. As shown in **Figure 2E**, the pull-down assays confirmed the dimerization of OR1 and OR2 with OR7. The conclusions from these experiments were further confirmed by bi-directional co-immunoprecipitation assays (data not shown). Thus, all available results corroborate previous reports on the role of OR83b family members as ubiquitous heteromerization partners of insect odorant receptors [5], [13], [14], [15].

### OR topology on the plasma membrane

**Figure 3:**
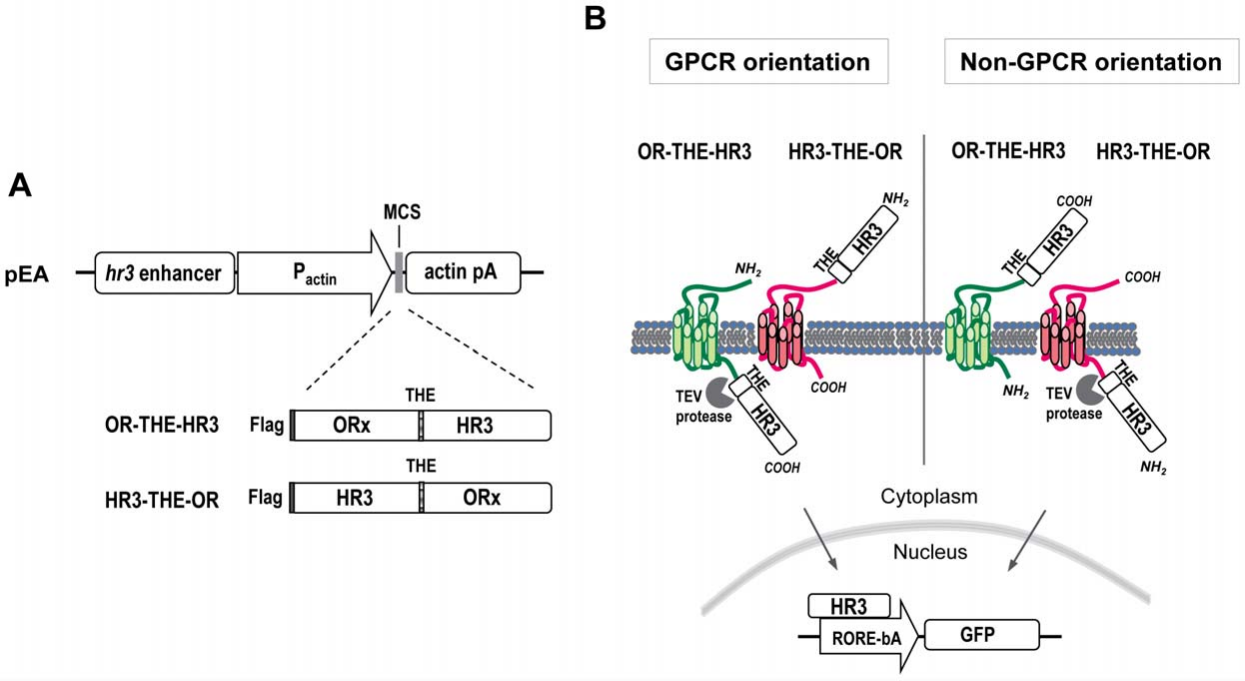
The “topology screen” assay. (**A**) Expression constructs. *hr3* enhancer, baculoviral (BmNPV) homologous region 3 enhancer sequence; pActin, *Bombyx mori* A3 cytoplasmic actin promoter; MCS, multiple cloning site; actin pA, 3′untranslated region of *B. mori* actin gene containing polyadenylation signals; Flag, epitope tag; OR; Odorant or opioid receptor ORF; THE, Tobacco etch virus protease recognition site; HR3, *Bombyx mori* hormone receptor 3. (**B**) Hypothetical model illustrating possible location of HR3 in both fusion constructs, with respect to the OR orientation (GPCR or not) in the membrane. RORE-bA, response element for retinoic acid receptor-related orphan receptor/basal actin promoter.

To deduce whether mosquito ORs have a GPCR-like topology on the cell membrane as mammalian ORs do or a *Drosophila*-like OR topology with their N-termini being located intracellularly, we developed a ‘topology assay’ that capitalized on the properties of the nuclear receptor HR3 of *B. mori* [36], [48], [49], [50], which is capable of activating gene promoters containing a *Drosophila* response element related to the mammalian retinoic acid receptor-related orphan receptor response element (RORE; [36], [48]). In this assay, the ORFs of the ORs under investigation are expressed as fusion proteins containing the HR3 factor at either their N- or the C-termini (**Figure 3A**). Interposed between the receptor and the transcription factor there is a polylinker sequence (‘THE’) encompassing a specific cleavage site for the Tobacco Etch Virus (TEV) protease [51], a 6xHis affinity purification tag and an EE detection tag. The chimeric receptor constructs were transfected in Hi5 cells together with a GFP reporter gene placed under the control of a RORE linked promoter. When the TEV protease is co-expressed in the cells, it recognizes and cleaves the site between the receptor and the HR3 factor only when the latter is located intracellularly but not when it is located outside the cell (**Figure 3B**). The resultant free HR3 then enters the nucleus and activates reporter gene transcription, which is monitored through the appearance of fluorescence in the transfected cells. Thus, by fusing the HR3 to the N- or the C-terminus of the ORs and co-expressing of TEV protease, the cellular location of HR3 and hence the orientation of the OR can be determined (**Figure 3B**).

**Figure 4:**
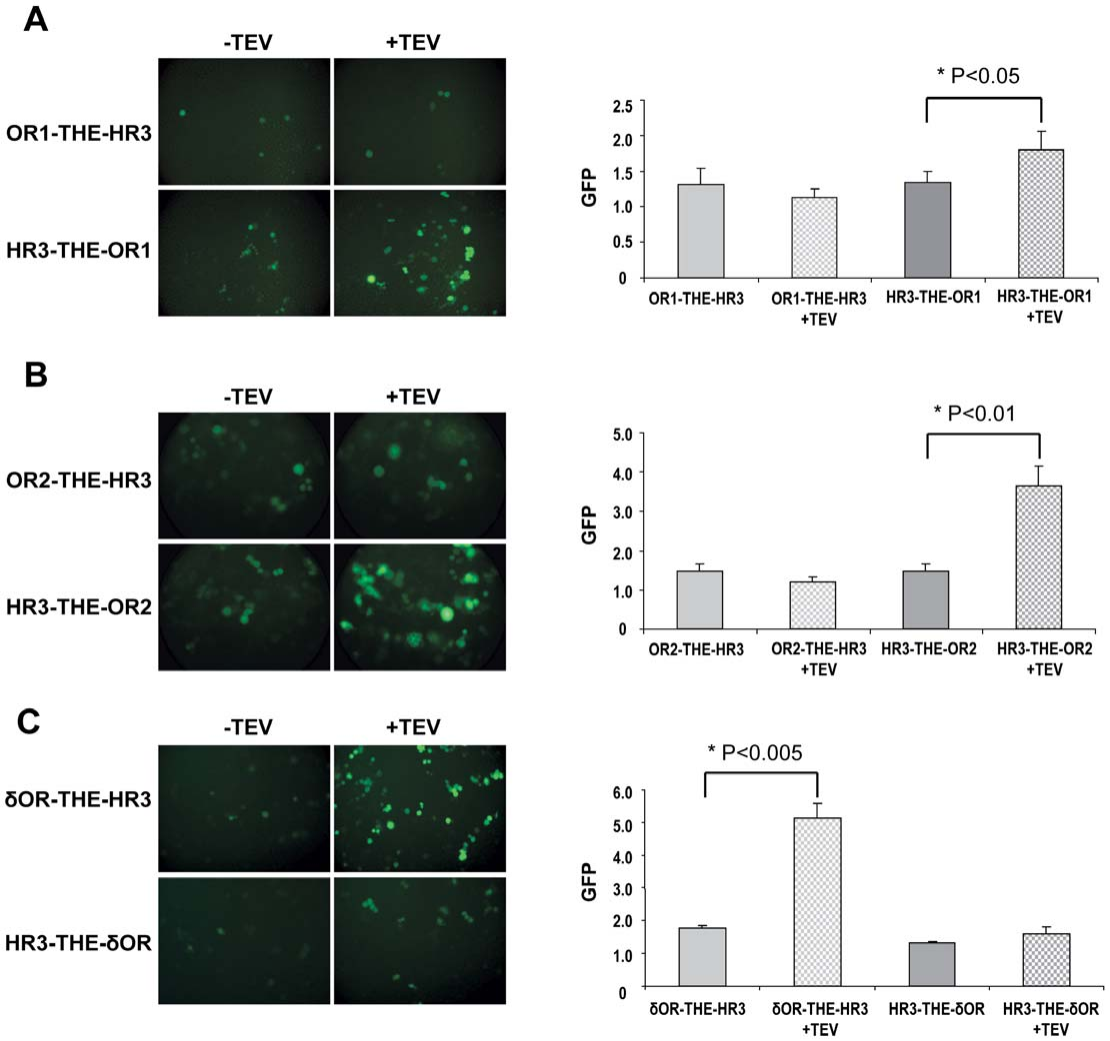
Topology assays for the mosquito OR1 and OR2. Chimeric receptor proteins fused at either their N- or the C- terminus to the TEV cleavage sequence (THE) and the HR3 transcription factor are co-expressed in Hi5 cells with a GFP reporter construct together with or without TEV protease. For both OR1 (**A**) and OR2 (**B**) N-terminal fusions (HR3-THE-OR1 and HR3-THE-OR2), expression of the TEV protease resulted in increase of fluorescence of the cells. No significant increase of fluorescence was detected when the C-terminal fusions of ORs were used (OR1-THE-HR3 and OR2-THE-HR3). Similar constructs of the human opioid receptor δ (δOR) that was used as control (**C**) give opposite results with an increased fluorescence for the C-terminal fusion (δOR-THE-HR3). For each chimeric receptor protein, both representative images (left) and quantitative results (right, with values representing the mean ± S.E.M. of four experiments) from the fluorometric analysis are shown.

As shown in **Figure 4A**, when OR1 was fused at its N-terminus to the HR3, expression of the TEV protease resulted in an increase of fluorescence of the cells, indicating release of HR3 and activation of the RORE-linked GFP. No increase of fluorescence was detected when the C-terminal fusion of OR1 was used. These results suggest that the N-terminus of OR1 is located intracellularly. Parallel analysis of the constructs for OR2 revealed that OR2 had an orientation identical to that of OR1 (**Figure 4B**). In contrast, equivalent fusions of the human δOR used as controls, gave opposite results (**Figure 4C**) indicating an extracellular N-terminus, consistent with the known structure of a member of the GPCR superfamily.

**Figure 5:**
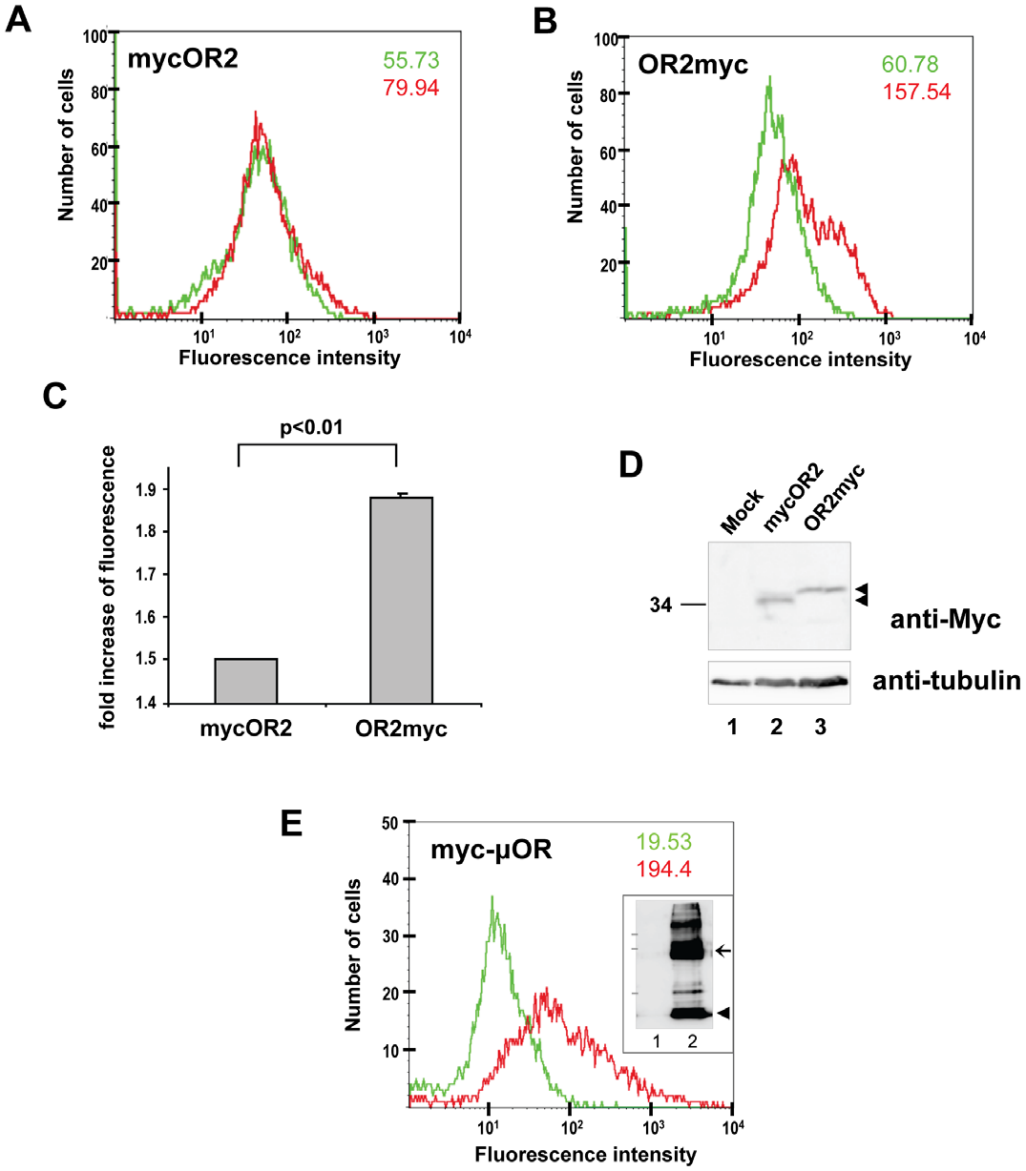
Flow cytometric analysis of expression of Myc-tagged OR2 on the surface of Bm5 cells. (**A**) N- or (**B**) C-terminally Myc-tagged OR2 were stably expressed in Bm5 cells in the presence of flagOR7 and analyzed by FACS for the extracellular localization of the Myc tag. For each panel, the green tracing and number represent fluorescence values obtained from the staining of the cells with only the FITC labelled secondary antibodies, while the red tracing and number represent values obtained from cells incubated with both the primary anti-Myc and the FITC labelled secondary antibodies. Increased fluorescence intensity (2.59-fold over the background value) was observed for the C-terminally Myc-tagged receptor in comparison to the receptor that was Myc-tagged at the N-terminal end (1.43-fold over the background value). (**C**) Values (increases over background) indicated in this panel represent the mean ± S.E.M. of three independent experiments. (**D**) Western blot analyses of whole cell lysates from the stable cell lines used for FACS analysis and control, mock-transformed cells, probed with anti-Myc (upper) and anti- tubulin (lower) antibodies. (**E**) FACS analysis of cells expressing the N-terminally Myc-tagged µ-opioid receptor used as a positive control for the extracellular localization of the Myc tag. Green and red tracing/numbers are as in panels A and B. Inset shows the detection of µOR in these cells by western blotting with the anti-myc antibody. The arrowhead and the arrow point to major bands detected (putative monomer and dimer, respectively), while the positions of 50, 90 and 118-kDa molecular mass markers are indicated at left.

To confirm the orientation of mosquito ORs on the plasma membrane by an independent approach, FACS analysis was performed on Bm5 cells stably expressing OR2 tagged with a Myc epitope at its N- or C-terminus in the presence of a co-expressed OR7. Because the cells were not subjected to any type of permeabilization or fixation, only extracellular tags could be detected by flow cytometry. This method has been used successfully in the past for the determination of the topological arrangement of the termini of membrane-anchored proteins on the plasma membrane [52], [53], [54], as well as the evaluation of the internalization of various GPCRs following different treatments [41], [55]. As can be deduced from the results shown in **Figure 5**, the level of fluorescence intensity was found to be much more pronounced for the C-terminally Myc-tagged receptor (**Figure 5B**) relative to the N-terminally tagged one (**Figure 5A, and 5C**), confirming the extracellular location of the C-terminus of the OR. Both cell lines appeared to express the relevant ORs at comparable levels (**Figure 5D**). Confirmation of the reliability of the detection method was obtained from the results of an identical FACS analysis carried out on Hi5 cells that had been transfected to express transiently a N-terminally Myc-tagged GPCR, the rat µ-opioid receptor (myc-µOR), used as a positive control for a “N-terminus out” topological orientation. As is evident from **Figure 5E**, a nearly10-fold increase in the fluorescent signal over background was obtained upon addition of the anti-myc antibody to the unfixed and non-permeabilized myc-µOR-expressing Hi5 cells. The significant difference in the signal to noise ratio between the transfected Hi5 cells, which expressed transiently myc-µOR (∼10-fold increase) and the stably transformed Bm5 cells expressing OR2myc (∼3-fold increase) is apparently due to the correspondingly different levels of Myc-tagged proteins expressed by the two cell populations.

## Discussion

In recent years, substantial progress has been made toward the functional characterization of many members of the OR family in flies and mosquitoes, particularly with respect to their function and the identification of ligands of natural origin that trigger their activation and downstream physiological responses *in vivo* and in a well-established *in vitro* system derived from *Xenopus laevis* oocytes [7], [9], [11], [12]. Despite this progress, however, the molecular and biochemical details of OR structure-function relationships that are responsible for the functional properties of these receptors, particularly mosquito ones, remain largely unexplored. Moreover, even though workers in the field have adopted readily the notion that mosquito ORs assume a *Drosophila* OR-like topology on the plasma membrane of expressing cells (N-terminus IN, C-terminus OUT; [5]), which is distinct from that of mammalian ORs and other 7TM domain receptors that signal through heterotrimeric G-proteins (reviewed in [56]), the notion had not been demonstrated formally through relevant experimentation.

In this study we successfully expressed three *A. gambiae* odorant receptors, ORs 1, 2 and 7 using a heterologous lepidopteran expression system, as a first step toward their biochemical characterization, which included the analysis of their localization in these cells and the establishment of their topology on the plasma membrane. Thus, efficient expression of various forms of the three mosquito ORs was achieved and documented by immunoblotting and immunofluorescence. The expression levels for any given receptor was not influenced to any significant degree by the various tags added to their termini or by the position of the tags.

Interestingly, while in *Drosophila* mutant neurons lacking OR83b, OR22a/b and OR43a were highly degraded with trace quantities detected only in the cell body [5], [57], we did not observe any enhancement of OR1 or OR2 protein levels upon co-expression of OR7, either in whole cell lysates or in the membrane fraction of the expressing cells. This difference from the *in vivo* findings could be due to a “masking effect” caused by the over-expression of the recombinant receptors in the specific expression system used or even the presence of an endogenous OR83b-like function in lepidopteran cells, as was reported to occur in another cell line, Sf9 [6]. As an additional comment we note that the recombinant ORs were found to migrate somewhat faster than expected on SDS gels, probably due to their highly hydrophobic nature [42], [43] or to detergent binding [58]. Moreover, a tendency for the mosquito ORs to aggregate after boiling, a rather common property for many membrane proteins [44], [59], was also observed in our studies (data not shown).

A potentially interesting finding of this study was the detection of polypeptides having mobilities in the denaturing and reducing gels used for our analyses suggestive of putative OR2 homodimers. Although the identity of the slower migrating species as dimers requires formal demonstration, we note that there have been numerous reports concerning the presence of SDS-resistant dimers for a variety of other receptors [45], [46], [47], mostly GPCRs for which it is well established that they function as dimers and/or higher order oligomers [60]. Moreover, as has also been pointed out previously [61], insect ORs are likely to undergo post-translational modifications that could affect many of their properties including stability, expression levels and internalization. Although a combination of approaches will be required for confirmation and interpretation of any results obtained, including the detected differences in expression levels between OR1 and OR2, which was consistently observed with different constructs, heterologous expression of insect ORs in cell culture systems, such as the one used in the present study, could prove to represent important tools for the dissection of the totally unexplored area of post-translational processing.

The topology of expressed mosquito receptors analyzed in this report has been determined through the use of a novel ‘topology screen’ assay capitalizing on the plasma membrane orientation-dependent cleavage of fusions of the membrane anchored ORs with the previously characterized orphan receptor of *B. mori* HR3 [36], [48]. This assay has the advantage of allowing for quantification, as opposed to the immunofluorescence approach [6], which relies on differences in protein detection efficiency in the absence or presence of detergent. Our findings from this ‘topology screen’ assay, as well as from the more classical FACS analysis, extend previous reports concerning the “N-terminus in/C-terminus out” topology of several *Drosophila* ORs [5], [6] to the mosquito receptors. More recently, while our work was in progress, the inverted topology of one lepidopteran OR, that of the light brown apple moth, *Epiphyas postvittana* has also been reported [62]. To our knowledge, however, this is the first report concerning topology of odorant receptors in mosquitoes, in general, and in this medically important disease vector, in particular. Moreover, we note that the ‘topology screen’ assay described here could be also used for the determination of the topology of the termini of other types of plasma membrane anchored proteins.

It is also important to note that our understanding of structure-function relationships for membrane proteins has lagged significantly behind that of soluble proteins, mainly due to the difficulties in expressing and purifying quantities of membrane proteins adequate for structural analysis. Recently, a synthetic human olfactory receptor, hOR17-4, has been heterologously over-expressed in mammalian cell culture and purified to almost 90% homogeneity [44], [63]. To our knowledge nothing similar has been achieved with an insect OR. Taking into account the advantages of lepidopteran insect cell culture systems, the expression system described here could prove useful for obtaining sufficient quantities for biochemical and structural characterization of mosquito ORs as well as ORs derived from other insect taxa and exploration of their functional properties.

An important issue that has yet to be addressed in a satisfactory fashion is the function of the mosquito ORs in the cultured insect cells, particularly in view of the pressing need for the development of high throughput screening (HTS) platforms that would allow the fast identification of mosquito OR ligands and ligand mimetics in collections of synthetic compound libraries and/or natural product secondary metabolites for use in rational mosquito control initiatives. Insect ORs have been reported recently to be ligand-gated cationic channels (ionotropic receptors) that may or may not encompass an additional cAMP-dependent metabotropic receptor component [14], [15]. Irrespective of the existence of the latter, a small number of papers have appeared, which reported on the detection of ligand-dependent stimulation in insect OR activity in insect and mammalian cell cultures using as probes fluorescent Ca^2+^ indicators [6], [62], [64], [65]. Although we have employed similar types of methodologies, our careful assessment of the results obtained from our own work and the data presented in the relevant literature reports suggests to us that the detection of ligand-dependent activation of the ORs using calcium influx changes may not be as robust as required for reliable quantitative reporting that could be applied toward HTS platform development for OR ligand mimetic discovery. Given the recent reports that demonstrate reliable measurements of ligand-dependent OR stimulation using clamp patching of *Xenopus laevis* oocytes injected with *in vitro* synthesized cRNA encoding mosquito ORs [7], [66], our aim is to develop analogous technologies for insect cells expressing recombinant mosquito ORs that are suitable for use in HTS formats. This work is currently in progress.
